# The Potential Role of Magnetic Resonance Spectroscopy in Image-Guided Radiotherapy

**DOI:** 10.3389/fonc.2014.00091

**Published:** 2014-05-05

**Authors:** Mai Lin Nguyen, Brooke Willows, Rihan Khan, Alexander Chi, Lyndon Kim, Sherif G. Nour, Thomas Sroka, Christine Kerr, Juan Godinez, Melissa Mills, Ulf Karlsson, Gabor Altdorfer, Nam Phong Nguyen, Gordon Jendrasiak, The International Geriatric Radiotherapy Group

**Affiliations:** ^1^Department of Psychology, Stanford University, Palo Alto, CA, USA; ^2^School of Medicine, University of Arizona, Phoenix, AZ, USA; ^3^Department of Radiology, University of Arizona, Tucson, AZ, USA; ^4^Department of Radiation Oncology, University of West Virginia, Morgantown, WV, USA; ^5^Division of Neuro-Oncology, Department of Neurosurgery and Medical Oncology, Thomas Jefferson University, Philadelphia, PA, USA; ^6^Department of Radiology, Emory University, Atlanta, GA, USA; ^7^Department of Radiation Oncology, Darmouth College, New Lebanon, NH, USA; ^8^Department of Radiation Oncology, Centre Val d’Aurelle, Montpellier, France; ^9^Department of Radiation Oncology, Florida Radiation Oncology Group, Jacksonville, FL, USA; ^10^Department of Radiation Oncology, University of Arizona, Tucson, AZ, USA; ^11^Department of Radiation Oncology, Marshfield Clinic, Marshfield, WI, USA; ^12^Department of Radiation Oncology, Camden Clark Cancer Center, Parkersburg, WV, USA; ^13^Department of Radiation Oncology, Howard University Hospital, Washington DC, USA; ^14^Department of Radiation Oncology, East Carolina University, Greenville, NC, USA

**Keywords:** MRS, IGRT, prostate cancer, GBM

## Abstract

Magnetic resonance spectroscopy (MRS) is a non-invasive technique to detect metabolites within the normal and tumoral tissues. The ability of MRS to diagnose areas of high metabolic activity linked to tumor cell proliferation is particularly useful for radiotherapy treatment planning because of better gross tumor volume (GTV) delineation. The GTV may be targeted with higher radiation dose, potentially improving local control without excessive irradiation to the normal adjacent tissues. Prostate cancer and glioblastoma multiforme (GBM) are two tumor models that are associated with a heterogeneous tumor distribution. Preliminary studies suggest that the integration of MRS into radiotherapy planning for these tumors is feasible and safe. Image-guided radiotherapy (IGRT) by virtue of daily tumor imaging and steep dose gradient may allow for tumor dose escalation with the simultaneous integrated boost technique (SIB) and potentially decrease the complications rates in patients with GBM and prostate cancers.

## Potential Advantages of Magnetic Resonance Spectroscopy for Functional Tumor Imaging

**Figure 1:**
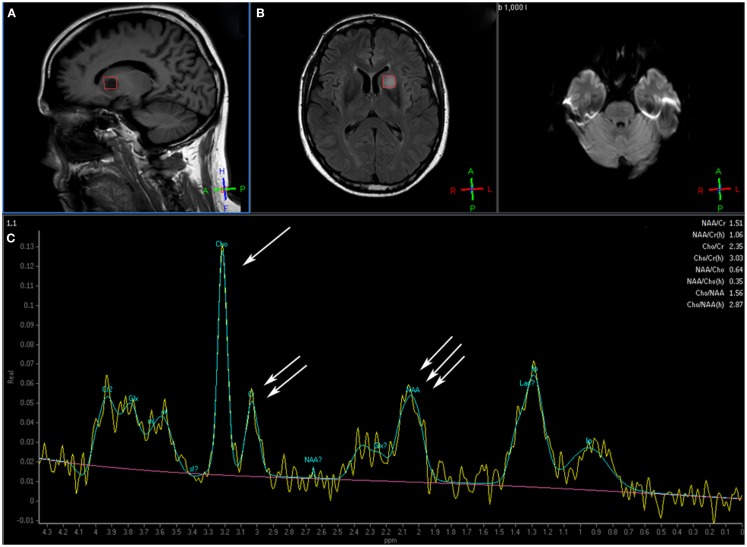
**This patient with a glioblastoma multiforme shows the voxel over the area of interest in the tumor over the sagittal T1 (A) and axial FLAIR (B) image**. Magnetic resonance spectroscopy **(C)** shows an elevated choline peak (single arrow) and decreased creatine (double arrow) and *N*-acetyl aspartate (NAA) peaks (triple arrow), which is the typical pattern for tumor (Images courtesy of Dr. Ashok Srinivasan, University of Michigan).

Magnetic resonance spectroscopy (MRS) is based on nuclear magnetic resonance technique to investigate the metabolism of chemicals in the body. Different chemicals containing the same nucleus exhibit characteristic chemical shifts in resonance frequency, allowing the chemical form of the element to be identified. Since the most abundant atom in the body is hydrogen (H), ^1^H MRS estimates the concentration of different metabolites within normal tissues of the body, which are displayed as a spectrum of resonances (peaks) along the *x*-axis as parts per million (ppm) and the amplitude of resonances is measured on the *y*-axis using an arbitrary scale. Depending on the clinical question, many major metabolites can be measured with MRS. In the brain, *N*-acetyl aspartate (NAA) is a marker for neuronal and axonal integrity. A decrease in NAA level is usually associated with neuronal loss or damage. Choline (Cho) represents the constituents of cell membrane. Increased Cho is associated with increased concentration of cells and or/cell membrane synthesis such as cancer. Creatinine (Cr) is a marker for cell energy metabolism. Decreased in Cr is associated with tissue death or necrosis. Lactate is a marker for anaerobic glycolysis. Increased lactate is associated with hypoxemia and tumors because of their anaerobic metabolism. Increased lipids concentration is observed in necrotic areas of the tumor. In gliomas, NAA is reduced because of neurons destruction by the tumor and Cho is increased because of tumor cell proliferation. Thus, abnormal Cho/NAA ratio is observed in areas of tumor infiltration such as the area of vasogenic edema around the gross tumor. Figure 1 illustrates the potential of MRS to outline the gross tumor volume (GTV) in a patient with glioblastoma multiforme (GBM).

**Figure 2:**
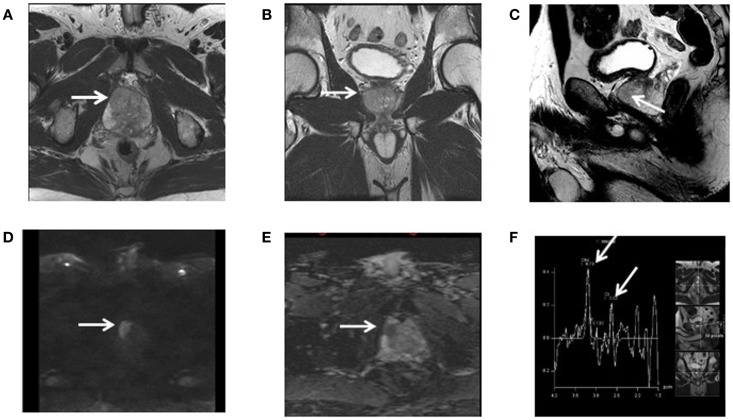
**This patient has a biopsy-proven adenocarcinoma of the prostate in the magnetic resonance spectroscopy area suspicious for malignancy**. Axial **(A)**, Coronal **(B)**, and sagittal **(C)** high-resolution T2-weighted images. Axial diffusion weighted, b = 2000 **(D)** and ADC map **(E)**. 1H-spectroscopy demonstrating elevated choline/creatine-to-citrate ratio **(F)**.

Magnetic resonance spectroscopy is particularly helpful to distinguish radiation injury from tumor recurrence after radiotherapy as both may have similar magnetic resonance imaging (MRI) appearances (1). Decreased in Cho, NAA, and Cr are usually observed with radiation injury and high Cho/NAA ratio is suggestive of tumor recurrence (2). In the prostate, Citrate (Cit) is produced by the normal prostate epithelium. The prostate has a high concentration of mitochondrial Zinc (Zn), which inhibits aconitase, the first enzyme of the Krebs cycle, which normally converts citrate to isocitrate leading to a high concentration of citrate in the prostatic epithelium (3). Cit is often decreased in area of prostate adenocarcinoma because of the low Zn concentration. High Cho and Cr are also observed in tumor areas because of cancer cells proliferation. Thus, higher Cho + Cr/Cit ratio is observed in areas of high tumor concentration compared to normal prostate tissue. Figure 2 illustrates the potential of MRS to outline the GTV in a patient with biopsy-proven adenocarcinoma of the prostate. Interestingly, areas of high Gleason score (4 + 3 or above) associated with tumor poor differentiation may be associated with high Cho + Cr/Cit ratio suggesting that MRS may be useful to guide prostate biopsy (4, 5). A high Cho + Cr/Cit ratio is also associated with a large tumor volume and advanced tumor stage (6, 7). As a result, MRS is very accurate to detect high grade tumors within the prostate gland, which may be useful for treatment planning because of the high recurrence rates of these tumors (8). As prostate cancer has a heterogeneous distribution within the prostate gland, MRS is particularly helpful to guide a second biopsy if the initial biopsy was negative among patients with a high PSA level suspicious for prostate cancer (9). MRS can also be used to assess radiotherapy response or recurrence following prostate irradiation. As Cit level decreases following prostate cancer irradiation at a faster rate than Cho or Cr, the Cho level, or Cho/Cr ratio are often used for radiation response.

A low normalized Cho following radiotherapy may predict a low PSA (0.5 ng/ml or less) at 1 year following prostate cancer treatment (10). Conversely, high Cho level or Cho/Cr ratio may detect local recurrences in patients with rising PSA following prostate cancer irradiation (11–13). The pattern of local recurrence following external beam irradiation of prostate cancer is predominantly within the dominant intra-prostatic tumors suggesting that radiation dose escalation of these focal tumor masses may improve local control (14).

## Principles of Image-Guided Radiotherapy

Conventional treatment with three-dimensional conformal radiotherapy (3D-CRT) has been associated with a higher rate of toxicity with radiation dose escalation because of irradiation of a large volume of normal tissues (15). The introduction of intensity-modulated radiotherapy (IMRT) has led to significant reduction of normal tissue irradiation because of the steep radiation dose gradient away from the target volume compared to 3D-CRT (16). However, a significant amount of normal tissues is still irradiated because the inclusion of the tumor and areas at high risk for invasion with a large rim of normal tissue called planning target volume or PTV to avoid marginal miss. Recently, image-guided radiotherapy (IGRT) by combining the steep dose gradient of IMRT with daily imaging may further improve treatment toxicity because of the PTV reduction provided that the gross tumor and area at risk for tumor invasion can be accurately outlined with proper diagnostic imaging (17). Functional tumor imaging such as MRS in combination with conventional diagnostic studies such as MRI may allow the radiation oncologist to develop a treatment plan that covers the PTV with a curative radiation dose while sparing the normal organs with the simultaneous integrated boost (SIB) technique, which delivers different dose levels within the PTV (18). Thus, the intra-prostatic or GBM GTV outlined by MRS may be treated with a higher radiation dose than the PTV for improved local control while the IMRT steep dose gradient decreases radiation dose to the normal adjacent organs and potentially reduces long-term complications. The success of functional imaging for accurate radiation delivery with IGRT requires a close collaboration between the diagnostic radiologist and radiation oncologist as MRS is not easy to interpret because of its limitations even for experienced diagnostic radiologist. As radiation dose escalation with IGRT may be associated with significant toxicity, it is imperative to outline the GTV accurately.

## Potential Application of MRS for IGRT of Glioblastoma Multiforme

Multiple studies of radiation dose escalation for GBM have failed to demonstrate an improvement in survival or local control (19). However, these studies were based on 3D conformal radiotherapy (3D-CRT) and MRI imaging. One possible explanation is whether the high radiation dose was actually delivered to the areas of high cancer concentration to be effective because of the heterogeneity of the tumor distribution within the target volume. Another possible explanation is the protracted treatment time may not be effective for local control because of the accelerated repopulation of cancer stem cells, which may be radio-resistant (20). Thus, a treatment course that delivers a high radiation dose to the GTV within a short time may be more effective for tumor control and potentially improve patient quality of life as they will have more time to spend with their family. Functional imaging with MRS may allow the radiation oncologist to outline the target volume accurately and spare the normal brain from unnecessary irradiation. The potential of IGRT to decrease the planning target volume (PTV) because of daily imaging coupled with the steep dose gradient of IMRT may further reduce treatment toxicity. As a result, the combination of precise tumor targeting and planning with MRS and effective radiotherapy delivery through IGRT may improve local control without excessive neurotoxicity. The feasibility of MRS for radiotherapy treatment planning of GBM has been investigated. The choline to creatinine ratio as an indice of the tumor activity (3 or higher) was converted on a gray scale, fused to the MRI images and transferred to the computer tomography (CT) scan as GTV in 12 patients with glioma (21). Among the patients in the study who had GBM, an IMRT plan was developed to delivered 5940 cGy in 180 cGy to the PTV while limiting radiation to the critical radiosensitive structures such as the optic chiasm and brain stem. Radiotherapy treatment was well tolerated by all patients without complications. A SIB plan was also generated but not used for treatment to increase the GTV dose to 7000 cGy based on the choline/creatinine ratio. Despite a higher GTV dose, the dose to the radiosensitive structures did not increase and highlighted the safety of radiation dose escalation with the SIB technique. Another study corroborated the feasibility of MRS for radiation dose escalation. Thirty-five GBM patients underwent surgical resection and had MRS to outline the tumor bed after surgery (22). The voxels within the post-operative T2 MRI that contained acholine/*N*-acetylaspartate ratio of 2 or above were treated with stereotactic radiosurgery (SRS) to a dose determined by the Radiation Therapy Oncology Group (RTOG) SRS guidelines followed by an additional dose of 6000 cGy with 3D-CRT. Among the 16 patients of the study who received temozolomide (TMZ) in addition to the radiotherapy protocol, median survival was 20.8 months compared to the historical control of 14.6 months for the ones treated with conventional radiotherapy and TMZ of the European Organization for Research and Treatment of Cancer (EORTC) (23). Grade 3–4 toxicity of the protocol treatment was acceptable suggesting that radiation dose-escalation based on MRS for GTV delineation may improve local control without excessive toxicity. Preliminary studies of MRI-based IMRT treatment of GBM also suggest that radiation dose escalation may be safe when combined with TMZ. Thirty-eight GBM patients were treated to a dose ranging from 6600 to 8100 cGy to the GTV with the IMRT technique. Only three patients with radiation dose exceeding 7500 cGy developed radionecrosis. Among the 22 patients who had functional imaging with C^11^ methionine positron emission tomography (MET–PET) before treatment, seven out of eight patients recurred because of inadequate coverage of the GTV as defined by MET–PET (24). This study highlights the potential of MRS to avoid marginal miss and possibly decreasing complications rates with PTV reduction. IMRT-based IGRT for GBM has been investigated to shorten the treatment course with promising results (25–27). Thus, MRS-based IGRT treatment for GBM merits further investigations in the future to improve local control and reduce toxicity.

## Potential Application of MRS for IGRT of Prostate Carcinoma

On line IGRT in prostate cancer allows for immediate correction of daily movement of the prostate secondary to bladder and rectum filling. The ability to increase radiation delivery accurately avoids unnecessary irradiation of the bladder and rectum and may decrease radiation side effects. In a study of 275 patients with prostate cancer treated to a tumor dose of 74–78 Gy, patients who had IGRT experienced significantly less diarrhea, urinary frequency, and fatigue compared to the ones without IGRT. The margins and planning constraints were the same for both groups (28). The reduced morbidity of radiation with daily imaging was also corroborated in another study of 282 patients with prostate cancer. Among 154 patients treated with IGRT, rectal pain and diarrhea were significantly less even though they were treated to a higher dose compared to the ones who did not have IGRT (29). Inpatients with intermediate to high risk prostate cancer, a higher radiation dose may be required to improve local control and biochemical-free survival if dose escalation does not lead to increased risk of complications (15). However, in patients with multiple co-morbidity factors such as the elderly, increasing radiation dose to the prostate may further increase the risk of rectal damage because the close proximity of the rectum to the prostate (30). Thus, increasing radiation dose to the intra-prostatic GTV may offer the ideal solution of limiting rectal dose while delivering a curative tumor dose. The feasibility of this treatment strategy was demonstrated in a dosimetric study of eight patients with prostate cancer (31). The intra-prostatic GTV as outlined with ^18^F Choline PET–CT, a cell proliferation marker with intense accumulation in prostatic cancer cell, was treated up to 90 Gy without exceeding the dose constraints to the bladder and rectum with the IMRT technique. Other dosimetric studies corroborated the feasibility of PET-based GTV dose escalation up to 100 Gy with IGRT for patients with prostate cancer (32, 33). The potential of MRS for potential intra-prostatic GTV dose escalation with IMRT was highlighted in one dosimetric study where the prostate was treated to 70 Gy at 1.8 Gy/fraction while the GTV was treated to 90 Gy at 2.25 Gy (34). Compared to an IMRT plan that conventionally treated the prostate to 70 Gy, the rectal dose was 40 and 48 Gy for the GTV dose escalation plan and conventional plan respectively. Thus, using MRS for potential GTV boost allows for a higher radiobiologic dose to the tumor while decreasing radiation dose to the rectum.

**Figure 3:**
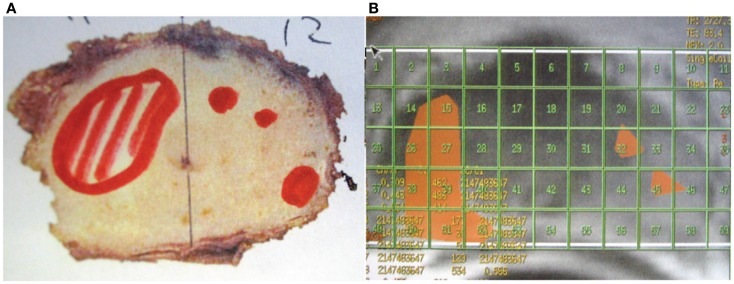
**This patient had a radical prostatectomy demonstrating two intra-prostatic adenocarcinoma in the pre-operative magnetic resonance spectroscopy areas suspicious for malignancy**. These two gross tumor volumes could have been treated to a higher radiation dose (81 Gy) while the prostate received only 78 Gy with the image-guided radiotherapy technique, thus improving the chance for local control and decreasing the risk of long-term rectal damage. **(A)** The areas outlined in red showed the cancerous tissue in the right and left lobe in the prostate. **(B)** The areas outlined in orange had an abnormal CCr (choline+creatine/citrate) ratio suspicious for malignancy on pre-operative magnetic resonance spectroscopy. (Images courtesy of Dr. Shigeo Horie, Teikyo University, and Dr. John G. Delinasios, International Institute of Anticancer Research.)

The safety of intra-prostatic GTV dose escalation was illustrated in a clinical study of 118 patients with intra-prostatic GTV defined either on MRI or MRS (35). The GTV and PTV were treated to 81–82 and 78 Gy, respectively with IMRT. No patient developed grade 3–4 gastrointestinal toxicity. Figure 3 illustrates the potential of MRS for GTV boost in a patient with prostate adenocarcinoma treated with radical prostatectomy (36). The patient had two intra-prostatic nodules suggestive of malignancy on pre-operative MRS and confirmed pathologically after surgery. These two GTV could have been treated to a high radiation dose to improve local control while sparing the rectum if the patient had definitive irradiation instead of surgery. In fact, this patient may be a good candidate for high dose rate prostate brachytherapy as these two foci can be treated to a higher dose by increasing the dwell time of the radioactive source (37). Preliminary clinical study of PET-based intra-prostatic GTV dose escalation with IGRT has been very promising because of minimal toxicity (38). As ^18^F-choline PET–CT may not be available in most centers, MRS-based GTV may be a practical method for IGRT dose escalation of prostate cancer. MRS may also play a significant role in the future because of its ability to detect recurrence following external beam prostate irradiation and for possible salvage (39).

## Limitations of Magnetic Resonance Spectroscopy

Multiple factors can potentially limit MRS. Inhomogeneities in the magnetic field can cause peak overlap and poor quantification. This can be limited if the magnetic field is shimmed prior to the MRS study, which helps to correct for magnetic field inhomogeneities. Susceptibility artifact can degrade the study if the area of interest is in a part of the brain close to bone or air, such as in the paranasal sinuses. Iron and other minerals built up in the paranasal ganglia can also cause susceptibility artifact that causes distorsion. In a similar fashion, if the spectrum is obtained in an area of the brain close to the scalp, scalp lipids can degrade the spectrum. Each box in an MRS exam called a voxel, is limited in spatial selectivity, and when using multivoxel MRS technique, there is a certain degree of overlap within each voxel from the adjacent voxels. In general, spatial resolution is also limited due to a low signal to noise ratio.

## Conclusion

Functional imaging with MRS may allow radiation dose escalation with IGRT for GBM and prostate carcinoma while sparing the adjacent normal organs. MRS should be integrated in future prospective studies to assess its potential to reduce long-term complications and possibly improving local control in patients with GBM and prostate cancers.

## Conflict of Interest Statement

The authors declare that the research was conducted in the absence of any commercial or financial relationships that could be construed as a potential conflict of interest.
